# *Stevia rebaudiana* germplasm characterization using microsatellite markers and steviol glycosides quantification by HPLC

**DOI:** 10.1007/s11033-021-06308-x

**Published:** 2021-04-03

**Authors:** Maria Margarida Ribeiro, Tatiana Diamantino, Joana Domingues, Ílio Montanari, Marcos Nopper Alves, José Carlos Gonçalves

**Affiliations:** 1 Centro de Biotecnologia de Plantas da Beira Interior, Escola Superior Agrária de Castelo Branco, 6001-909 Castelo Branco, Portugal; 2 Instituto Politécnico de Castelo BrancoEscola Superior Agrária, 6001-909 Castelo Branco, Portugal; 3 Centro de Recursos NaturaisAmbiente e Sociedade (CERNAS) - Instituto Politécnico de Castelo Branco, 6000-084 Castelo Branco, Portugal; 4 Forest Research Centre, School of Agriculture, University of Lisbon, Tapada da Ajuda, 1349-017 Lisbon, Portugal; 5 CPQBA/UNICAMP - Centro Pluridisciplinar de Pesquisas Químicas Biológicas e Agrícolas, Universidade Estadual de Campinas, São Paulo, Brazil

**Keywords:** Fingerprint, Genetic improvement, Germplasm, Stevioside, Molecular markers, Rebaudioside A

## Abstract

*Stevia rebaudiana* Bertoni, Asteraceae, is an herbaceous perennial plant native to Paraguay. This species is considered since ancient times a medicinal plant with important bioactive compounds and pharmacologic and food properties, namely diterpenes glycosides. The high natural sweetener potential stevioside and rebaudioside A produced by *S. rebaudiana* plants are suitable sucrose substitutes, and their obtention is influenced by environmental, phytosociological, and genetic factors. The plants’ genetic profile and sweet potential depiction are needed for suitable plant selection for improvement and deployment. Thirty-one *S. rebaudiana* accessions grown in the same plot where leaves samples were collected in early 2019, were genotyped using six microsatellite markers, including two steviol glycosides biosynthesis functionally involved markers. Additionally, an aqueous extract of each sample was obtained in a water bath and purified by SPE for stevioside and rebaudioside A quantification by normal phase HPLC. Stevioside and rebaudioside A contents varied between 0.53–7.36% (w w^−1^) and 0.37–3.60% (w w^−1^), respectively. Two genotypes displayed interesting ratios of rebaudioside A/stevioside (number 3 and 33). The level of genetic similarity between genotypes was tested through a pairwise similarity coefficient, and two groups of individuals had the same fingerprinting. Strong relatedness was found within genotypes, possibly due to cloning, thus, influx of new germplasm ought to be made to prevent mating between relatives, and for further selection and genetic improvement.

**Supplementary Information:**

The online version contains supplementary material available at 10.1007/s11033-021-06308-x.

## Introduction

The genus *Stevia* consists of approximately 230 species [1, 2], and *Stevia rebaudiana* features the sweetest essence [3]. *Stevia* genus is distributed worldwide, ranging from the southern parts of the USA to Argentina and Brazil. Nowadays, *Stevia* cultivation has spread worldwide, including Europe, propagated through seed and cuttings [2]. *S. rebaudiana* Bertoni (2n = 22) is a perennial and branched shrub of the Asteraceae family, native to north-eastern Paraguay [4, 5]. Since ancient times, this plant has been known as a sweetener due to the high content of diterpene glycosides [6], found in the leaves (present in a minor amount in shoots, roots, and flowers) in concentrations from 4 to 20% of dry matter [5, 6]. Stevioside and rebaudioside A are the interesting steviol glycosides sugar-substitutes substances, which tend to accumulate in the aging tissues, thus, older leaves contain more sweeteners than younger ones. Conversely, at the beginning of flowering, the concentrations of glycosides in the leaves begin to decrease [1]. In general, stevioside is found in higher amounts than rebaudioside A, thus both substances' production is negatively correlated. Rebaudioside A is a better commercial product than stevioside, due to higher solubility in water and sweeter capacity, and the latter is responsible for aftertaste bitterness [1, 7]. According to Ceunen and Geuns [8], this ratio can be influenced by ontogeny and day length, with larger ratios obtained during the vegetative stage under short days. Rebaudioside A/stevioside ratio in leaves, together with the steviol-glycosides concentration and the leaf dry yield are significant economic parameters for stevia genotypes evaluation [9]. Moreover, in the former study, the ratio of rebaudioside A to stevioside revealed a significant effect of the genotype, in a trial conducted with two genotypes. An interesting approach to overcome the bitter aftertaste and improve the *Stevia* sweetness potential is the biotransformation of stevioside into rebaudioside A. Adari et al. [10] explained the stevioside enzymatic transglycosylation performed via the *Stevia* leaves pre-treatment with cellulose. The steviol-glycosides are currently used as sweet-tasting non-caloric food additives, due to obesity and diabetes rates rise worldwide [4, 7, 11]. Furthermore, displays therapeutic properties, such as antimicrobial, anti-inflammatory, antioxidant, antiviral, anti-jaundice, cardiotonic, and diuretic [7, 12]. The leaves and extracts can be taken raw or cooked, and added to various food products as sweeteners, due to the steviol-glycosides thermostability [13]. Steviol-glycosides were approved for use as a food additive in Europe represented by the code E960, in 2011 [14]. The existence of genetic variability in plant genetic resources provides plant breeders with the possibility to develop new and improved cultivars with desirable characteristics to include stakeholders-preferred traits (e.g., [15]).

Some genetic studies were reported in this species using markers with several limitations, such as RAPDs [16–18], AFLPs, and ISSRs [19–21], due to their dominance and to RAPDs reproducibility problems [22]. Dominance leads to recessive allele frequency underestimation biasing genetic diversity estimates [23, 24]. Thus, for fingerprinting purposes, microsatellite markers (SSRs) are the markers of choice, owing to their high polymorphism, multiallelism, codominance, high reproducibility, and uniform distribution in the genome [25–27]. These markers can competently be used in the genotyping process with a good genome sampling, which makes them suitable to estimate genotypes relatedness that requires multilocus genotype identification [28]. Kaur et al. [29] and Bhandawat et al. [30] were the pioneers in microsatellite markers development and use in *S. rebaudiana* and, recently, from freely available expressed sequence data (ESTs), and Cosson et al. [31] also developed molecular markers (EST-SSRs) for population genetic and germplasm characterization studies. Recently, also a group of *S. rebaudiana* genotypes from Paraguay were studied with SSR and ISSR [32], and analysed as dominant markers. Additionally, molecular data can be pooled with biochemical data to detect stevia genotypes suitable to start a breeding program [18].

The use of microsatellite loci and the development of statistical tools made it possible inferring kin relationships from molecular data, in particular ‘relatedness’, meaning the estimation and assignment of pairs or groups of individuals to categories of relationship or a measure of the fraction of alleles shared identical by descent among individuals [33]. Indeed, to ensure that levels of co-ancestry and inbreeding among selected plants are kept to a minimum, it is advantageous to know their relatedness [34]. Additionally, besides avoiding related individuals (in particular for outcrossed species), it is important to accurately identify the genotypes and to distinguish the new cultivars for registration purposes. The germplasm fingerprint and cultivar identification with molecular markers-based grew in importance, due to generated data quality and speed, and also because morphological characteristics are influenced by environmental factors [22, 35–37].

In addition to the genetic and environmental factors that can influence the steviol-glycosides content, the analytical methodology selection is important to obtain an efficient protocol for plant bioactive compounds present extraction, purification, and quantification. The *Stevia* leaves steviol-glycosides were generally obtained subsequently to hot water application, followed sometimes by solid-phase extraction. However, extraction methods using other solvents, such as ethanol and methanol or even supercritical fluid extraction, were also described [38]. These solvents, namely methanol, appear to be used in the extraction process, presumably to improve extraction efficiency and facilitate the individual steviol-glycosides separation. However, regarding industry food safety, the use of water for extraction should be favoured over methanol or ethanol use [11]. Additionally, the water extraction seems the extraction method to be used in this case, given that rebaudioside A water solubility is higher compared to stevioside [11]. Several HPLC methodologies used to quantify the steviol-glycosides in *S. rebaudiana* leaves samples were reported in the literature [38 and references therein, 39]. Matrix load dramatically shortens chromatographic columns’ lifetime, thus the analytical purification method, including solid phase extraction (SPE), must be applied [7, 38]. Finally, the HPLC methods was considered as the most reliable and simple method for the quantification of glycosides [40].

The genotypes collection of *S. rebaudiana* established in the Pluridisciplinary Center for Chemical, Biological and Agricultural Research, State University of Campinas, São Paulo, Brazil (CPQBA/UNICAMP) were studied for breeding purposes. The aims of the present study were: (i) to genotype 31 individuals with microsatellite markers to compute the genetic diversity, the fingerprint, and the relatedness, and (ii) to identify the genotypes that produce the highest rebaudioside A/stevioside ratio.

## Material and methods

### Plant material

We have studied 31 individuals from the *S. rebaudiana* germplasm plot established in the campus of the CPQBA/UNICAMP (Pluridisciplinary Center for Chemical, Biological and Agricultural Research. State University of Campinas, São Paulo, Brazil). The *S. rebaudiana* seeds were previously collected in the species’ region of origin, but the exact location is unfortunately unknown. Four to five leaves sampled in each genotype from different parts of the plant were collected from January to February 2019. The genotypes were grown in the same plot with 1 × 0.5 m spacing, and 5 to 10 replications per genotype and the sampling was made just before flowering. Afterward, the leaves were lyophilized and frozen at – 80 °C.

### DNA extraction and amplification

DNA was extracted from 100 mg of fresh leaves of each *S. rebaudiana* genotype, using the CTAB method as described by Doyle and Doyle [41], and a high concentration of pure genomic DNA was obtained. The genomic DNA from all individuals was amplified using six microsatellites (SSR) markers selected from literature (Table 1). The two selected SUGMS (*Stevia* UniGene derived MicroSatellites) primers (Table 1) are putatively related to the steviol biosynthesis [30]. All amplifications were conducted separately for each primer pair and forward primers were 6-FAM fluorescently labelled. PCR reactions with the SUGMS primers (Table 1) were performed in 10 µL a total reaction volume, containing 50–60 ng of template DNA, 0.2 U Supreme NZYTaq 2× Colourless Master Mix® separate MgCl_2_ (Nzytech, Lisbon, Portugal), 2.5 mM MgCl_2_, and 1.0 μM of each primer. The amplifications were performed on a UNO96 Gradient thermocycler (VWR®, Leuven, Belgium). The PCR protocol consisted of an initial denaturation step of 4 min at 94 °C, followed by 35 amplification cycles composed of denaturation (1 min at 94 °C), annealing (1 min at optimal annealing temperature for each pair, see Table 1) and polymerizing (1 min at 72 °C). After the amplification cycles, a final extension step was performed for 7 min at 72 °C.

**Table 1:** Primers used for the SSRs analysis

Primer	Sequence 5′–3′	Size range (bp)	Annealing temp. (°C)	References
SUGMS28	F: CAAATTGGGAATTGCAGCTT	210–310	55	Bhandawat et al. [30]
	R: GACAAACAAGCCGAGAGAGG			
SUGMS43	F: CCAATCTACAATTGCCACAAGA	225–255	55	Idem
	R: TTTTCCGAGGTTTTTGGTTG			
gi18465444	F: ATGAAAGCGAGCCTGATGAT	100–610	56	Kaur et al. [29]
	R: TCAAGCAACGATTCTTTCCA			
gi16949765	F: CAAGGCTTGCTCCGAAATAC	680–900	56	Idem
	R: TCATCTGCAAGTGCTTCCTC			
gi18465673	F: CGGGTTAGAAGGAAACGTGA	500–800	56	Idem
	R: AAGTTTCCACCAACCCATCA			
stvia036	F: TGTCTCTGACAAAATTTATACGG	144–180	55	Cosson et al. [31]
	R: TTGTCTGTCACCCTGTGG			

The PCR reactions with the ‘gi’ primers (Table 1) were performed in a 10 µL total reaction volume, containing 50–60 ng of template DNA, 0.8 U Supreme NZYTaq 2× Colourless Master Mix® separate MgCl_2_ (Nzytech, Lisbon, Portugal), 2.5 mM MgCl_2_, and 1.5 μM of each primer. The amplifications were performed in the same thermocycler, programmed with an initial denaturation step of 5 min at 95 °C, followed by 40 amplification cycles composed of denaturation (1 min at 95 °C), annealing (1 min at optimal annealing temperature for each pair, see Table 1) and polymerizing (2 min at 72 °C). The PCR reaction with the stvia036 primer was performed in a 10 µL total reaction volume, containing 50–60 ng of template DNA, 0.5 U Supreme NZYTaq 2 × Colourless Master Mix® separate MgCl_2_ (Nzytech, Lisbon, Portugal), 2.5 mM MgCl_2_, and 2.0 μM of each primer (Table 1). The amplifications were performed in the same thermocycler, programmed with an initial denaturation step of 15 min at 95 °C, followed by 35 amplification cycles composed of denaturation (30 s at 94 °C), annealing (45 s at 55 °C, see Table 1) and polymerizing (1 min at 72 °C). After the amplification cycles, a final extension step was performed for 5 min at 72 °C. All PCR products were diluted in 50 μL of Milli-Q water and an aliquot of 3.0 µL of each dilution was mixed with 10 µL of formamide and 0.2 µL of LIZ-600 size standard. Genotyping was performed with an ABI 3130 Genetic Analyzer (Applied Biosystems, Foster City, CA, USA), the fragment analysis was performed using GeneMapper 4.0 software (Applied Biosystems, Foster City, CA, USA), and the data manually scored.

### Aqueous extracts of *S. rebaudiana*

Lyophilized leaf samples were grounded with a mortar and pestle. Sample extracts were prepared by weighting 0.4 g of dried leaf powder and adding 8 mL of distilled water into 15 mL centrifuge tubes, afterward mixed in the vortex and placed in a 90 °C water bath for 30 min. Subsequently, the extracts were cooled at ambient temperature and centrifuged at 4400 rpm for 30 min at 4 °C (CLINIconic 75003623, Thermo Scientific, Massachusetts, EUA). From each centrifuged extract, the supernatant was transferred to a 25 mL volumetric flask. The pellet was reused for two more extractions and the supernatants transferred to the same volumetric flask. Finally, the volumetric flask was filled to 25 mL with distilled water.

### Stevioside and rebaudioside A extracts purification and HPLC quantification

Both steviol-glycosides were quantified according to the improved HPLC method described by Woelwer-Rieck et al. [38], with some modifications. The extracts were purified two times by solid-phase extraction (SPE) with the C18-E column (100 mg, 1 mL, 55 µm, 70 Å, Strada, Phenomenex, California, USA), with a constant vacuum pressure of 15 inHg. Columns were first conditioned with 3 mL of methanol and 3 mL of Milli-Q water and subsequently charged with 400 µL of extract, followed by washing with 3 mL of Milli-Q water and 5 mL of acetonitrile: Milli-Q water (20:80 v/v). Columns were left to dry for 3 min and then, steviol-glycosides were eluted with 2 mL of acetonitrile:Milli-Q water (75:25 v/v) and recovered in a 5 mL volumetric flask.

Seven stevioside (Cayman Chemical, Michigan, USA) and rebaudioside A (Extrasynthese, Genay, France) standard solutions were prepared in acetonitrile:Milli-Q water (75:25 v/v) in the concentration range of 5–800 µg mL^−1^. Both standard solutions and purified extracts were filtered with a 0.45 µm nylon filter (0.4 mm diameter, Technocroma, Barcelona, Spain) before loading in HPLC.

The linearity was assessed by injecting standard solutions of stevioside and rebaudioside A from 10 to 800 µg mL^−1^, using six different concentrations, and all standard solutions were injected in two different days. Standards were loaded in triplicate and each purified extract in duplicate into HPLC (Agilent 1100 series, with a quaternary pump, autosampler, diode array detector, degasser, thermostat, and data system), equipped with an NH_2_ column (Purospher® STAR NH_2_, 250 × 4 mm, 5 µm) under the following conditions: 20 µL of injection volume, 210 nm wave-length, mobile phase: HPLC grade acetonitrile:Milli-Q water (75:25 v/v), 1 mL min^−1^ constant flow rate, 20 min at 36 °C. The qualitative determination was achieved by comparing the stevioside and rebaudioside A standard solutions retention times with those of the samples. The quantification was possible by applying the computed calibration curves equations. The linear regression curves of stevioside and rebaudioside A are presented below were obtained (both adjusted the interception in 0):

$${\text{Stevioside: Y = 4}}{\text{.7091 X }}\left( {{\text{r}}^{{2}} { = 0}{\text{.9994}}} \right)$$

$${\text{Rebaudioside A: Y = 4}}{\text{.1668 X }}\left( {{\text{r}}^{{2}} { = 0}{\text{.9998}}} \right)$$

where Y is the peak area (mAU s^−1^) and X is concentration (µg mL^−1^) (unpublished results). The stevioside and the rebaudioside A concentration were represented in % w w^−1^ (steviol glycoside/dry leave).

### Genetic data analysis

The genetic diversity parameters Na (number of alleles), Ne (effective number of alleles), Ho (observed heterozygosity), He (unbiased expected heterozygosity), and F (fixation index) were obtained with the GenAIEx software version 6.502 [42]. To test the genotypes pairwise relatedness the Lynch and Ritland [43] kinship coefficient was used (hereafter LR), and this coefficient was selected for it was considered the most accurate in a simulation with a set of relatedness coefficients validated with an elite group genetic similarity of *Eucalyptus globulus* with known pedigree [44]. The dendrogram (UPGMA method) was performed using the kinship matrix and the software NTSyS-PC version 2.11 [45]. The genetic relationship among genotypes was further analyzed by principal coordinate analysis (PCoA) using GenAIEx, based on the same pairwise relatedness matrix. The maximum deviation method was used for the factorial rotation and the main components were extracted, with the first main component and the second main component being the leading coordinates. The LR pairwise matrix, the genotypes multilocus comparison analysis, were computed in the GenAlEx.

## Results

### SSRs analysis

The analysis of the genetic diversity parameters (Table 2) revealed that the number of alleles per locus (Na) ranged from 3 to 6, averaging 4 per locus. The effective number of alleles per locus (Ne) was 3.02, lower than the average Na, but reflecting more accurately genetic diversity. Indeed, three loci displayed the same Na (3) and two had higher Ne than the other one (gi16949765), also reflected in higher genetic diversity values: the SUGMS43 and Stevia36 loci (Table 2).

**Table 2:** Genetic diversity parameters by locus for the 31 genotyped *S. rebaudiana* individuals

Locus	Na	Ne	Ho	He	F
**gi18465444**	**5**	**4.06**	**0.84**	**0.77**	− 0.11
gi16949765	3	1.60	0.45	0.38	− 0.21
**gi18465673**	**6**	**3.59**	**0.87**	**0.73**	− 0.21
SUGMS28	4	3.42	0.77	0.72	− 0.09
SUGMS43	3	2.66	0.84	0.63	− 0.34
Stvia36	3	2.81	0.81	0.65	− 0.25
Mean	4	3.02	0.76	0.65	− 0.20
SE		0.35	0.06	0.06	0.04

Values in bold correspond to primers with the highest genetic diversity parameters: Na, Ne, Ho and He

*Na* number of alleles, *Ne* effective number of alleles, *Ho* observed heterozygosity, *He* expected unbiased heterozygosity, *F* fixation index

However, the loci with higher Na, in general, had higher Ne and Ho values (gi18465444 and gi18465673), which were found in putative non-coding regions, values in bold in Table 2. However, the SUGMS43 locus, found in the coding region, had appreciable genetic diversity (Ho = 0.84), though with only three alleles, yet with even frequencies (data not shown).

The total observed heterozygosity (Ho) ranged from 0.45 to 0.87, with an average of 0.76. There was an excess of heterozygous in all loci since the observed is higher than that expected heterozygosity (Ho > He), while a lower difference is found in the SUGMS28 locus (Table 2). However, the analyzed germplasm is a group of individuals unlikely in Hardy–Weinberg equilibrium.

### Genetic distances and clustering

**Fig. 1:**
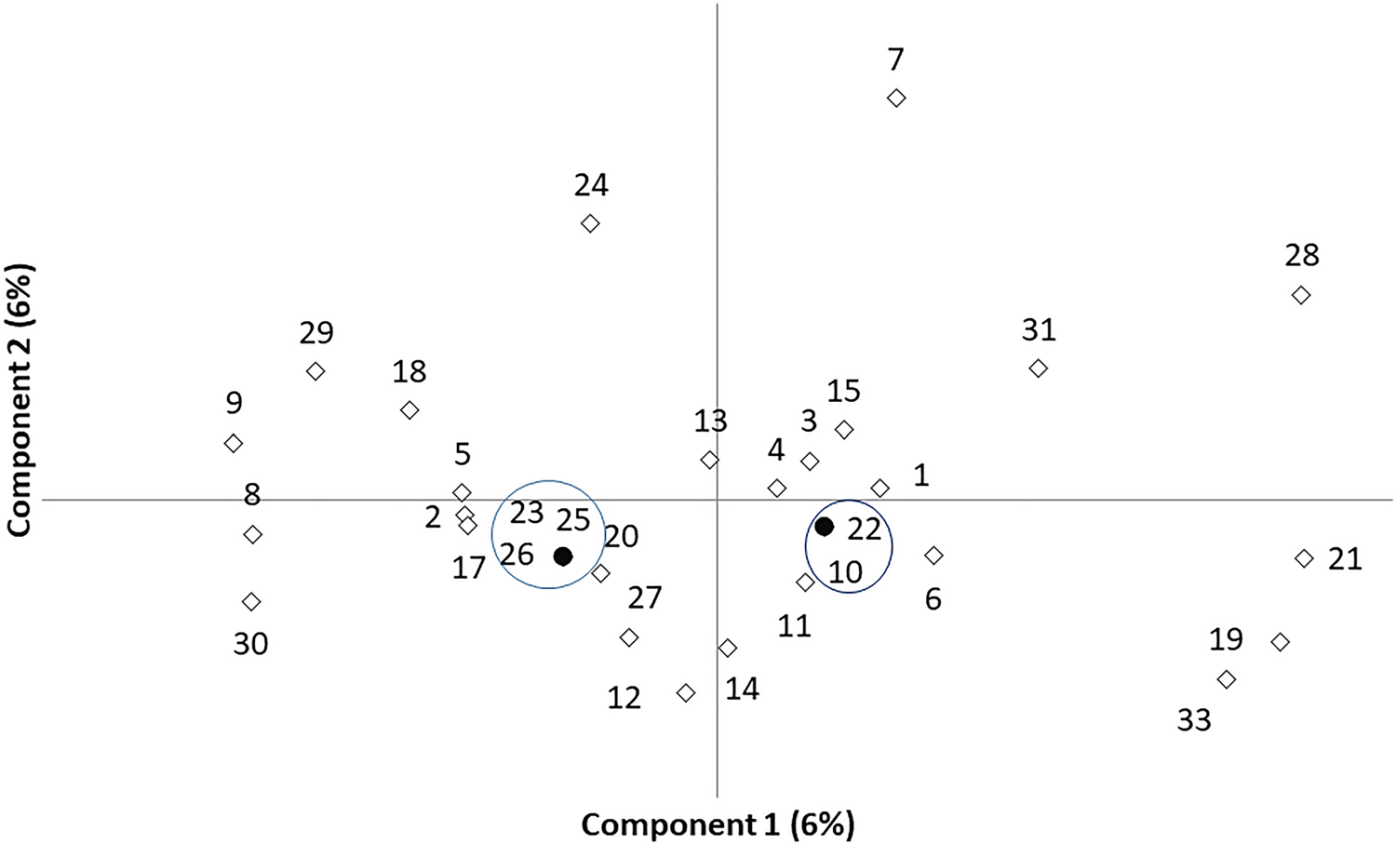
The principal component analysis was based on the LR pairwise kinship matrix. Black circles embodied individuals with the same genotype, all the others were represented by white diamonds. Both the first and the second components account for 6% of the total variation

Genotypes were grouped according to the level of genetic similarity using the LR index. In the PCoA analysis, based on the kinship distance (LR), individuals with the same genotype plotted together (black circles, Fig. 1), but the remaining ones were, in general, well individualized, except in the case of genotypes 2, 5 and 17. Both the first and the second component explained ≈ 6% of the total variation and the total variation explained was 12%.

**Fig. 2:**
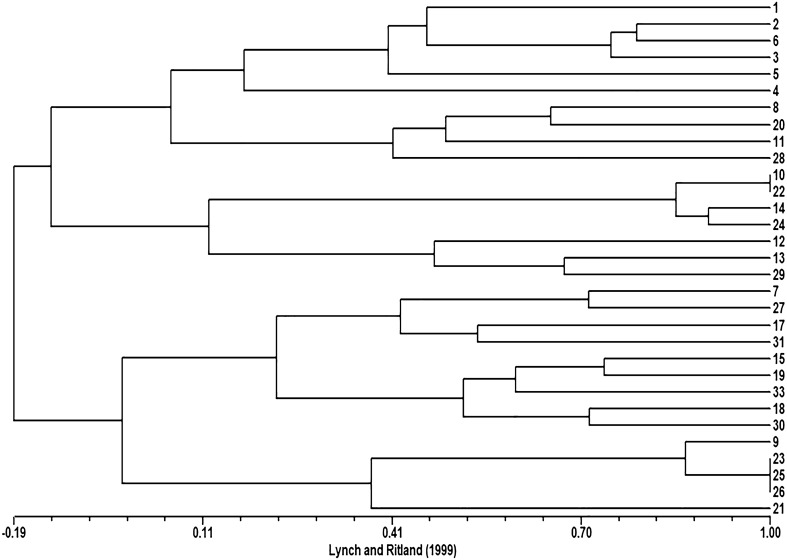
Accessions’ dendrogram using the UPGMA clustering methods and the Lynch and Ritland [43] pairwise kinship matrix

The dendrogram (UPGMA method) obtained with the kinship matrix (LR) (Fig. 2) reveals the relatedness among *S. rebaudiana* genotypes. This analysis separated the genotypes into two groups, with a very low degree of similarity (group 1: 4, 7, 9, 15, 17, 18, 19, 21, 23, 25, 26, 27, 30, 33, and 31; group 2: 1, 2, 3, 4, 5, 6, 8, 10, 11, 12, 13, 14, 20, 22, 24, 28 and 29). According to the literature for a value of LR coefficient > 0.25 the genotypes may be considered half-sibs, resulting from cross-fertilization; for LR > 0.5 the genotypes may be full-sibs, sharing both mother and father, for LR > 0.67 they may be the result of self-pollination, and for LR = 1 for the same genotype or clones [33, 44]. Indeed, two sets of individuals shared the same multilocus genotype (1st set: 10 and 22, and 2nd set: 23, 25, and 26), meaning that they share the same genotype, possibly a sampling/labeling error or clones. The fingerprinting of the two sets that displayed the same genotype are listed in Table S1.

### HPLC quantification of steviol-glycosides

The contents of stevioside and rebaudioside A in the different genotypes were determined using calibration curves obtained by authentic standards. The linearity corresponds to the method’s ability to provide results directly proportional to the analyzed compound concentration, within a given interval. The calibration curves were linear in the range of 10–800 µg mL^−1^ for both steviol glycosides standards and the linearity of the HPLC method was excellent (r^2^ > 0.99).

**Fig. 3:**
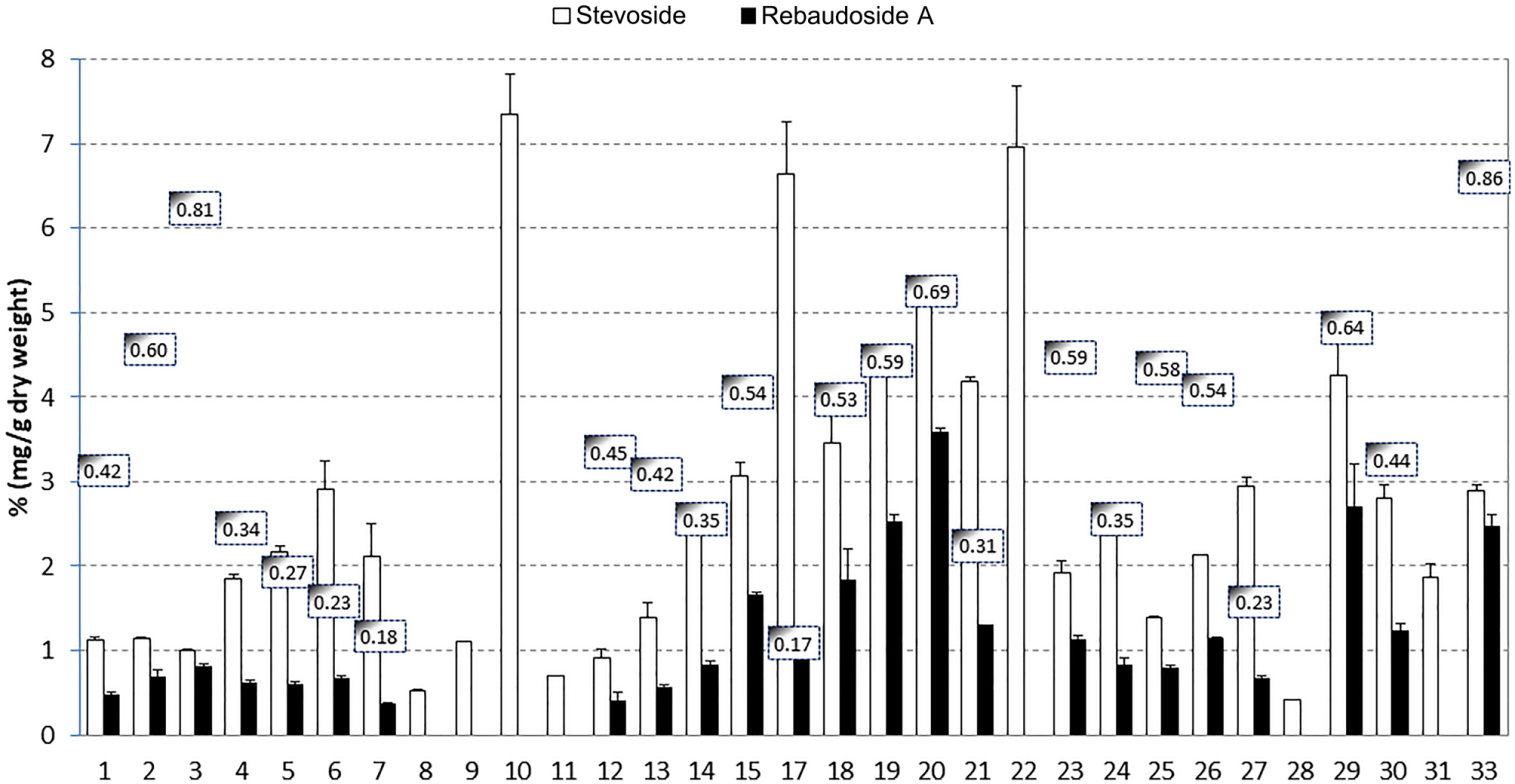
Stevioside (white bars) and rebaudioside A (black bars) content (% w w^−1^ dry leaf). White labels show the *S. rebaudiana* genotypes rebaudioside A/stevioside ratio. Two genotypes had the highest ratio level (3 and 33)

The stevioside and rebaudioside A retention time (RT) were 5.28 ± 0.05 min and 6.91 ± 0.10 min (mean ± standard deviation) respectively, with the HPLC method. Figure 3 shows the stevioside and rebaudioside A mean concentrations (% w w^−1^ dry leaf) for each sample and the respective rebaudioside A/stevioside ratio. The rebaudioside A-concentrations were lower in all the genotypes compared to the stevioside ones. From the results, significant among sample differences existed in both steviol glycoside concentrations. Samples 10, 17, and 22 had the highest stevioside content (6.64–7.36%), while samples 19, 20, and 29 had the highest rebaudioside A value (2.53–3.60%). Samples 8, 11, and 28 showed a lower concentration of both steviol glycosides compared to the other samples. Several samples revealed undetectable rebaudioside A content (8, 9, 10, 11, 22, 28, and 31), considering the HPLC conditions linearity method. The rebaudioside A/stevioside ratio should be as high as possible, given the rebaudioside A better sweetening properties than stevioside, as previously referred. The ratio ranged from 0.17 to 0.86, and the highest rebaudioside A/stevioside ratio was observed in samples 3 and 33, rating 0.81 and 0.86, respectively (Fig. 3). Decreasing ratio values were found in samples 2, 20, and 29, with 0.60, 0.69, and 0.64, respectively.

## Discussion

The *S. rebaudiana* is undergoing domestication [31], notwithstanding that the species was used as a sweetener as earlier as the 1940s, and cultivated worldwide [1 and references therein]. Nevertheless, few studies reported both the species genetic information and the steviol glycosides content [31 and references therein], and no study stated both the genotypes’ genetic relatedness and their fingerprint. In this study, microsatellite markers were used to reveal the genetic diversity and the genetic relationship among the 31 individuals analyzed and to further detect highly similar genotypes. In the study made by Bhandawat et al. [30] the authors used 40 selected genotypes with a set of SUGMS markers and they found that the observed diversity was significantly higher than the expected one (0.80 and 0.63, on average, respectively), similarly to the results from the current study (0.76 and 0.65). In both studies, the expected diversity was slightly lower than the observed one, resulting in a negative fixation index. But the plants used in the two studies had different origins making a genotype collection improbably in Hardy–Weinberg equilibrium. Possibly this could result in a “melting pot”, meaning a mixture of individuals from different origins, and putatively with very different allelic compositions [46]. Simultaneously, the average He value (0.78) obtained by Cosson et al. [31], was higher than the value from the current study, but the authors surveyed a much larger germplasm set (145 genotypes) with a wider origin range and a high number of markers. In a meta-study surveying species with different mating systems and using nuclear microsatellite markers to evaluate genetic diversity, the observed and expected estimates were, on average, 0.65 and 0.63, respectively [23], for the group of outcrossed species. The high level of genetic variability obtained in *S. rebaudiana* is most likely due to allogamy in this species mating system [31]. Moreover, the use of an artificial population constituted by individuals from different origins could explain a high value of observed genetic diversity in our study.

Estimators are useful to identify relatives and to minimize consanguinity in breeding populations in the absence of pedigree information [44]. Different relatedness estimators exist in the literature and they respond differently to the available sample [47]. The Lynch and Ritland [43] coefficient (LR) was selected as the appropriate kinship estimator in this study, as it proved to be impartial, accurate, with a low percentage of overlapping values between kinship groups and low percentiles of exact confidence [44]. The genotypes were initially divided into two groups and distributed according to the level of similarity in the dendrogram. Considering the values of LR, two sets of individuals had the same genotype, possibly for they were the same individual due to cloning (LR = 1.0). Vegetative propagation was used to maintain the collection, and mislabeling is possible in these cases (Ílio Montanari Jr., personal communication). In the other cases, the LR values grouping the genotypes was very high, detecting appreciable relatedness in the group of individuals studied. Indeed, cultivar mislabelling is possible [36], and the rapid development of DNA-based molecular markers are helpful to overcome this problem.

The steviol-glycosides content and variability determination are important for the plant producers and industries to obtain performant cultivars through selection and breeding. Therefore, all individuals from the current study were grown in the same conditions and collected just before flowering, when steviol-glycosides content is expected to be highest. The steviol-glycosides concentrations in the current study varied between 0.53–7.36% (w w^−1^) for stevioside, and 0.37–3.60% (w w^−1^) for rebaudioside A. In some samples, the rebaudioside A remained undetected, probably due to either steviol-glycoside production being lower than needed for quantification by the linearity HPLC conditions or to the genotype lack of production. In general, the steviol-glycosides concentrations observed in the thirty-one samples are following the values found in the literature, with stevioside content always higher than the rebaudioside A content [7, 11, 40]. Woelwer-Rieck et al. [38] reported that plants growing in a different cultivated field also showed a higher stevioside content than rebaudioside A values, with mean values of 7.90% (w w^−1^) and 4.93% (w w^−1^), respectively. Similar steviol-glycosides values were also obtained in another study, by water extraction, with stevioside values ranging between 3.84 and 9.36% (w w^−1^), and the rebaudioside A values ranging between 2.59 and 7.77% (w w^−1^) [48].

Despite the glycosides great solubility in water, some studies reported extraction with other solvents (e.g., [11]). Methanolic extracts had a range 4.10–8.20% (w w^−1^) and 0.60–4.44% (w w^−1^) of stevioside and rebaudioside A, respectively [39]. Analogous values have been reported in hydroethanolic extracts with a range of stevioside and rebaudioside A concentration between 3.78–9.84% (w w^−1^) and 1.62–7.27% (w w^−1^), respectively [9]. Regardless of the steviol-glycosides absolute quantity, the quality is defined by a high rebaudioside A/stevioside ratio. Indeed, the rebaudioside A/stevioside ratios obtained in the current study are under the numbers reported in the literature, with values ranging from 0.34 to 0.77 [9, 38]. Actually, in this study two genotypes showed interesting levels of rebaudioside A/stevioside ratio, the genotypes 3 and 33, with 0.81 and 0.86, respectively. In wild plants, this ratio is less than 0.5 [1]. Besides, those two genotypes were found in the two different clusters of the dendrogram, with very low LR, and they are unrelated individuals.

Comparing the genetics with the chemical results, it is worth noting that individuals 8, 11, and 28 revealed close genetic relatedness and similar chemical profile, with low steviol-glycosides concentration. Individuals 10 and 22 had displayed the same genetic fingerprint and the same chemical profile, both in stevioside concentration and absence or very low rebaudioside A synthesis. Individuals 23, 25, and 26 had the same fingerprint, and they also revealed a very similar chemical profile, being probably the same genotype/clone. Indeed, since the late 1980s, DNA fingerprinting has become an immensely important instrument for genotype identification in wild plant and cultivated species. Additionally, when the morphological characters are mostly quantitative, correspondence with DNA marker estimates is generally quite high as compared to qualitative characters, which are more likely to reflect only a small number of mutation events [37]. But, due to this group of individuals’ high relatedness, a new influx of unrelated genotypes should be included in this collection to further improve breeding and selection and avoiding relatedness. Additionally, other studies should be undertaken to reveal, e. g., genotypes’ leaves biomass production and other plant characteristics that would increase steviol-glycosides production (see Ref. [1] for details).

## Supplementary Information

Below is the link to the electronic supplementary material.

Supplementary file1 (DOCX 17 kb)

## Data Availability

All the data and material will be available upon request to the authors.
