# Supplementary material for: Stevia rebaudiana germplasm characterization using microsatellite markers and steviol glycosides quantification by HPLC
Source: Mol Biol Rep. 2021 Apr 3;48(3):2573–82. doi: 10.1007/s11033-021-06308-x (PMC8060219; doi:10.1007/s11033-021-06308-x)
Supplement: Supplementary file 1 — Supplementary file1 (DOCX 17 kb) [file 11033_2021_6308_MOESM1_ESM.docx]

**Supplementary material**

Ribeiro MM, Diamantino T, Joana D, Montanari Jr. I, Alves MN, Gonçalves JC

*Stevia rebaudiana* germplasm characterization using microsatellite markers and steviol glycosides quantification by HPLC. Molecular Biology Reports.

MM Ribeiro (corresponding author)

Instituto Politécnico de Castelo Branco,

Escola Superior Agrária, Castelo Branco, Portugal.

[mataide@ipcb.pt](mailto:mataide@ipcb.pt)

+351-272339900

**Table S1** List of sample pairs with matching multilocus genotypes

| **Sample** | **gi18465444** | | **gi16949765** | | **gi18465673** | | **SUGMS28** | | **SUGMS43** | | **Stevia36** | | |
| --- | --- | --- | --- | --- | --- | --- | --- | --- | --- | --- | --- | --- | --- |
| 10 | 80 | 87 | 26 | 33 | 32 | 47 | 29 | 32 | 26 | 34 | 139 | 153 |  |
| 22 | 80 | 87 | 26 | 33 | 32 | 47 | 29 | 32 | 26 | 34 | 139 | 153 |  |
|  |  |  |  |  |  |  |  |  |  |  |  |  |  |
| 23 | 72 | 85 | 33 | 33 | 27 | 38 | 29 | 38 | 26 | 30 | 147 | 153 |  |
| 25 | 72 | 85 | 33 | 33 | 27 | 38 | 29 | 38 | 26 | 30 | 147 | 153 |  |
| 23 | 72 | 85 | 33 | 33 | 27 | 38 | 29 | 38 | 26 | 30 | 147 | 153 |  |
| 26 | 72 | 85 | 33 | 33 | 27 | 38 | 29 | 38 | 26 | 30 | 147 | 153 |  |
| 25 | 72 | 85 | 33 | 33 | 27 | 38 | 29 | 38 | 26 | 30 | 147 | 153 |  |
| 26 | 72 | 85 | 33 | 33 | 27 | 38 | 29 | 38 | 26 | 30 | 147 | 153 |  |
